# Variations in compositions and antioxidant activities of essential oils from leaves of Luodian *Blumea balsamifera* from different harvest times in China

**DOI:** 10.1371/journal.pone.0234661

**Published:** 2020-06-16

**Authors:** Yuan-Hui Wang, Ya-Ru Zhang

**Affiliations:** School of Food Science and Technology, Henan University of Technology, Zhengzhou, China; ICAR- Indian Agricultural research Institute, INDIA

## Abstract

Xanthoxylin was the main compound (content 44.92% of total volatiles) in the leaves of Luodian *B*. *balsamifera*, which might be the key cause of failure in collecting essential oil (EO) of the leaves using general hydrodistillation in Clevenger apparatus. A modified hydrodistillation equipped with Clevenger apparatus was designed for isolating EO from the leaves. Six EOs of Luodian *B*. *balsamifera* harvested once a month from September to next February were collected successfully. The main components of EOs were *δ*-elemene, *α*-cubenene, caryophyllene, caryophyllene epoxide, *γ*-eudesmol, xanthoxylin, and *α*-eudesmol. The EOs of Luodian *B*. *balsamifera* collected from October to December had higher antioxidant activities (ACs). Combining the principal component analysis of chemical components with the results of ACs and the yields of six EOs, the leaves of Luodian *B*. *balsamifera* were suitable to be harvested in November and December to obtain EO with high quality.

## Introduction

*Blumea balsamifera* (L.) DC. (Asteraceae) is an aromatic plant and a traditional herb cultivated in East Asia and Southeast Asia [1,2]. The leaves of *B*. *balsamifera* are rich in volatile compounds and can be used to collect essential oil (EO), and EO is found to possess antimicrobial and insecticidal activities [3–6]. Besides, the crude extracts, flavonoids, sesquiterpenoids, and triterpenoids were extracted from the leaves of *B*. *balsamifera* [7–12]. *B*. *balsamifera*, also named Ainaxiang, harvested from Yunnan and Hainan Province, China, had been used to extracted EOs using hydrodistillation according to the method noted in Pharmacopoeia of the People’s Republic of China, and the compositions and antioxidant activities of EOs of different plant organs from *B*. *balsamifera* at different growth times were analysed and evaluated [13]. As is well known, the same species of plants cultivated in different regions have great differences in chemical composition [14]. *B*. *balsamifera* is a genuine Miao nationality herb which is cultivated in Luodian County, China, and is locally called as “Qian Ainaxiang” [15], and Luodian *B*. *balsamifera* is an important source of *l*-borneol [16]. The volatile oil of Luodian *B*. *balsamifera* had been extracted by hydrodistillation and organic solvent extraction in previous researches [17,18]. However, no information about the EO of Luodian *B*. *balsamifera* collected using Clevenger apparatus has appeared on the relevant investigation.

The preparation method of EO has been strictly defined, and the accurate specification is recorded in European Pharmacopoeia [19]. Clevenger apparatus is the classical laboratory equipment based on the circulatory distillation approach for collecting EO [20]. It must be noted that the entire distillate (hydrolate and oil) is not extracted with a solvent. Otherwise, the crude product cannot be called any longer an EO, it is just a solvent extract from the distillate [21]. The previous literatures recorded that organic solvent was used in the distillation process of Luodian *B*. *balsamifera* for collecting the extracts [15,17], therefore, the product was called a solvent extract rather than EO, including the remains recovered using some solvent from the walls of the glassware (Clevenger apparatus). The solvent-free extraction of EO is a green preparation process and is worth to be investigated. In our previous work, the EO of Luodian *B*. *balsamifera* leaves was collected unsuccessfully by the ordinary hydrodistillation in Clevenger apparatus. However, *l*-borneol was present on the inner wall of the condenser in the hydrodistillation, and the purity of *l*-borneol was 82% [22]. The above results can be used as a reference, and a modified hydrodistillation method was designed using volatile oil extraction apparatus (VOEA) recorded in the Chinese Pharmacopeia to collect EO of Luodian *B*. *balsamifera* leaves [23].

In the study, we investigated the reason why we cannot obtain the EO by the general hydrodistillation using Clevenger apparatus more deeply. Moreover, the EOs of Luodian *B*. *balsamifera* leaves harvested from different times were collected using Clevenger apparatus by the modified hydrodistillation method. The chemical components of EOs were determined by GC-FID and GC-MS, the antioxidant activities of EOs were evaluated by DPPH radical scavenging test, *β*-carotene bleaching (BCB) test, and thiobarbituric acid reactive species (TBARS) assay. Moreover, the optimum harvest time of Luodian *B*. *balsamifera* leaves was reported.

## Material and methods

### Plant material

The leaves were randomly collected from *B*. *balsamifera* cultivated in planting base (the planting base belongs to Gui Zhou Ai Yuan Eco-Pharmaceutical Development Co. Ltd) in Luodian County (Southwest China, 25° 04′ N; 106° 28′ E), and the samples were collected once a month from September 2016 to February 2017. The plant was identified by Prof. Y.N. He (Institute of Biotechnology, Guizhou Academy of Agricultural Sciences, China), and a voucher specimen (CGA-Dafengai-Guizhou-2009-11) was deposited in the Institute of Biotechnology, Guizhou Academy of Agricultural Sciences. The leaves were air-dried in the room and then were milled into 80 mesh powder before hydrodistillation.

### Chemicals

*n*-Hexane (99%), *l*-borneol (97%), xanthoxylin (97%) and 1,1-diphenyl-2-picrylhydrazyl (DPPH, 95%) were purchased from Sigma-Aldrich (Shanghai, China). *β*-carotene was purchased from Fluka Chemie (Buchs, Switzerland). Thiobarbituric acid, anhydrous Na_2_SO_4,_ and all of the applied solvents (analysis purity) were purchased from Sinopharm Chemical Reagent Co. Ltd. (Shanghai, China). For Linear retention indices (RIs) determination, a hydrocarbon mixture (C_8_–C_30_ *n*-alkanes) purchased from Supelco (Ballefonte, USA) was used to perform in the same condition as real sample determination.

For the measurement of response factors, the chemicals used were: *l*-borneol, ledol, *γ*-eudesmol, *β*-eudesmol, 1-octen-3-ol, linalool, nerolidol, 3-octanol and *α*-terpineol for alcohols; pentadecanal for aldehydes; camphor, zierone and xanthoxylin for ketones; hexadecanoic acid for acids; *β*-pinene for monoterpene hydrocarbons; *α*-muurolene, *δ*-cadinene caryophyllene and *α*-caryophyllene for sesquiterpene hydrocarbons; caryophyllene epoxide for oxides; 1,2-dihydro-1,1,6-trimethyl-naphthalene for aromatic hydrocarbons.

### Extraction of total volatiles

Fifty grams dried leaves of *B*. *balsamifera* were distilled to collect 5 L of distillate (distillation time: 10 h), and the distillate was collected for extracting total volatiles (TV) of the leaves using same volume anhydrous diethyl ether under room temperature. The extract solution was dehydrated with anhydrous sodium sulfate and concentrated to obtain total volatiles. The TV was stored at –20°C in dark glass bottles until required. The process was executed in ten replicates.

### Hydrodistillation

**Fig 1:**
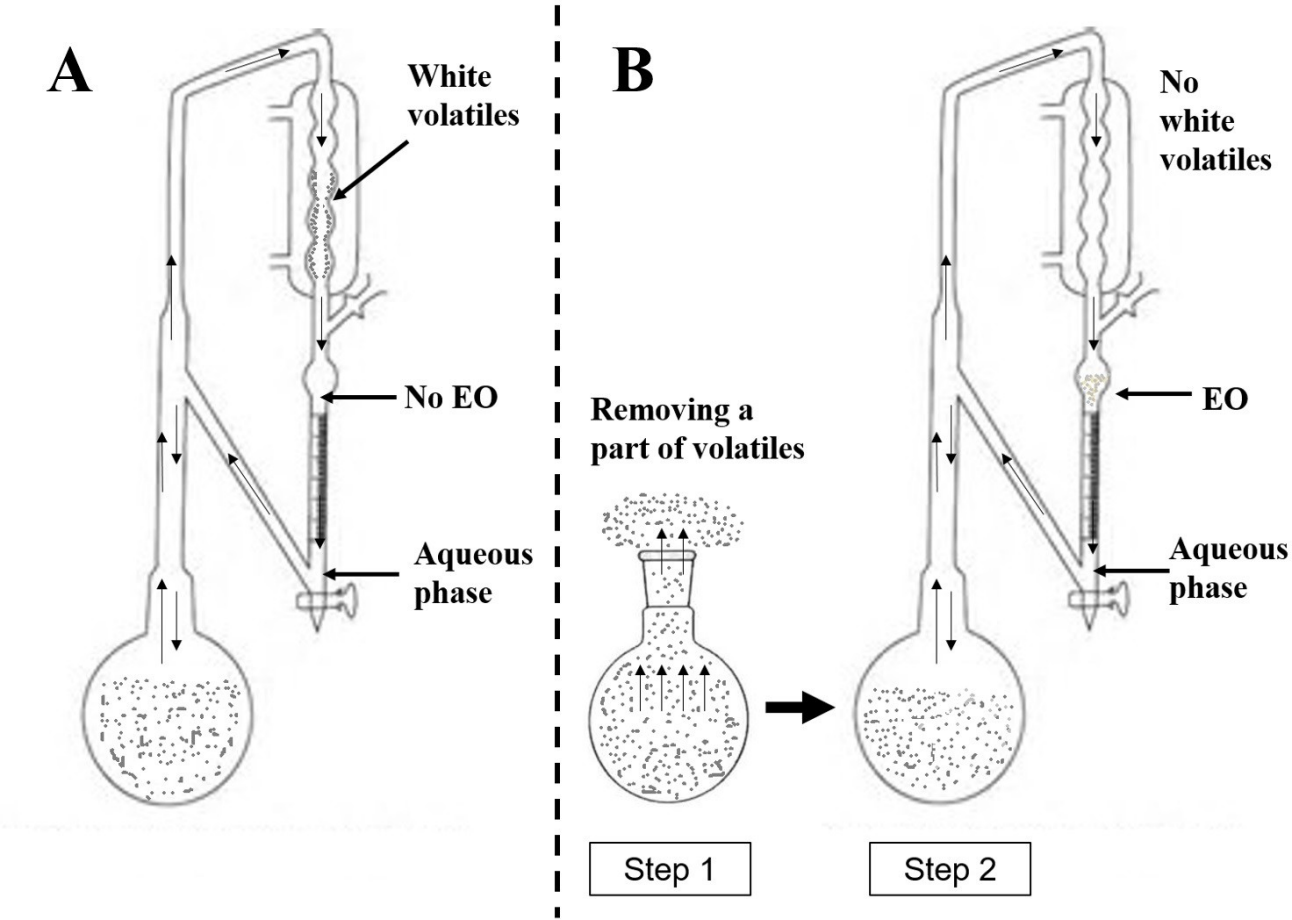
Clevenger apparatus: (A) unsuccessful hydrodistillation; (B) successful modified hydrodistillation procedure.

The general hydrodistillation was executed in Clevenger apparatus. Fifty grams of leaves samples and 1 L distilled water were added into 2 L flask and distilled for 10 h in Clevenger apparatus (Jingbo, Jintan, China) (Fig 1A). In the end, there was no EO to be collected. However, there were white volatile on the surface of condenser inner wall and an aqueous phase (aromatic water) in Clevenger apparatus. The aqueous phase was extracted by diethyl ether anhydrous (100 mL), and the extraction solution was concentrated under vacuum. White volatiles (WV) and the extract of aqueous phase (EAP) were weighed and stored at 4°C in dark glass bottles until analysis, respectively. The experiments were executed with six replicates.

### Modified hydrodistillation procedure

Fifty grams of leaves samples and 1 L distilled water were added into 2 L flask. At first, the mixture was heated to boiling temperature, and 500 mL distillate was collected and then extracted using diethyl ether anhydrous (500 mL), the extraction solution was concentrated to obtain the extract which was named “Aifen” (AF). The residual mixture was added with 500 mL of deionized water and then connected to the Clevenger apparatus for being distilled continuously. In the end, two phases were observed in Clevenger apparatus, an EO and an aqueous phase (Fig 1B). The EO was collected and dried with anhydrous Na_2_SO_4_. The yield of EO was used as a reference to optimize the distillation time (from 2 h to 10 h). AF and EO were stored at 4°C in dark glass bottles until analysis. The processes were executed with ten replicates.

### Gas chromatography (GC)

The oil samples for GC analysis were prepared in 99% *n*-hexane. The final concentration was 0.5 mg/mL. GC analysis was executed on a Shimadzu GC-2010 gas chromatograph operated with a split/splitless injector and a Shimadzu AOC-20i autoinjector (Shimadzu, Kyoto, Japan). Column: CP-WAX capillary column (30 m × 0.25 mm × 25 μm) (Varian, USA) and DB-5 capillary column (30 m × 0.25 mm × 25 μm) (Agilent, USA). Temperature program: from 45°C (1 min) to 280°C (3 min) at 5°C/min. Injection temperature: 250°C. Injection volume: 1.0 μL. Carrier gas: Nitrogen, flow rate: 3 mL/min. Injection mode: split (1:5). FID temperature: 250°C. H_2_ flow: 47 mL/min; air flow: 400 mL/min. Data handling was carried out by means of GCsolution 2.3 (Shimadzu).

The internal standard method was used in the quantitative determination of *l*-borneol and xanthoxylin and was performed on GC-FID system mentioned above. Naphthalene was used as the internal standard (IS). The samples and IS were prepared in ethyl acetate, and the final concentration was 0.2 mg/mL. The validation of quantitative determination as follows: the linear range was 0.6–1875 μg/mL for *l*-borneol and xanthoxylin; the accuracy was verified by eight replicate analyses with standard solution, the calculated RSD values for *l*-borneol and xanthoxylin were 0.16% and 0.21%, respectively; moreover, the recoveries of two compounds were 97% and 96%.

### Gas chromatography—mass spectrometry (GC-MS)

GC-MS analysis was executed with a Varian 1200L GC-MS/MS model gas chromatograph-mass spectrometer equipped with an autoinjector (Varian, Walnut Creek, CA, USA). Two capillary columns were the same as GC-FID system. The temperature program: from 45°C (0.5 min) to 280°C (3 min) at 5.0°C/min. Injection temperature: 250°C. Injection volume: 1.0 μL. Injection mode: 1:10. Carrier gas: He, constant linear velocity: 1.0 mL/min. MS interface temperature: 280°C; MS mode: EI; detector voltage: 1 kV; mass range: 35–500 uma; acquisition mode: full scan.

### Identification of constituents

Individual compound was identified by the following two methods, (1) mass spectrum was compared with those reported in NIST and WILEY libraries, Adam’s records and those of literature data; (2) Linear RIs relative to (C_8_–C_30_) *n*-alkanes were used in the identification of chemical components and matched with those of our own authentic compounds, Adam’s records and literature data.

### Quantification of constituents

The quantitation of EO components was based on the peak area normalization with response factors (RFs). The constituents of EO were complex, and these compound standards were lack, so it was unrealistic to determine the RFs of all compounds. Therefore, we used a solution introduced by Costa *et al*. [24], based on EO constituents classified by their functional groups and chemical classes. RFs are the average values of the response factors measured by individual standard compounds within the same chemical class and are recorded in Table 1. The quantification was applied in the concentration determination (%) of all oil components.

**Table 1:** Response factors (RFs) of different compounds.

Compounds	Mean ± STD	RF
***Alcohols***		
*l*-Borneol	1.30 ± 0.01	1.31 ± 0.01
Ledol	1.34 ± 0.02	
*γ*-Eudesmol	1.30 ± 0.01	
*β*-Eudesmol	1.30 ± 0.01	
1-Octen-3-ol	1.34 ± 0.02	
Linalool	1.31 ± 0.01	
Nerolidol	1.34 ± 0.01	
3-Octanol	1.34 ± 0.02	
*α*-Terpineol	1.26 ± 0.01	
***Aldehydes***		
Pentadecanal	1.30 ± 0.01	1.30 ± 0.01
***Ketones***		
Camphor	1.29 ± 0.01	1.30 ± 0.01
Zierone	1.31 ± 0.02	
Xanthoxylin	1.30 ± 0.01	
***Acids***		
Hexadecanoic acid	1.55 ± 0.02	1.55 ± 0.02
***Monoterpene hydrocarbons***		
*β*-Pinene	1.04 ± 0.02	1.04 ± 0.02
***Sesquiterpene hydrocarbons***		
*α*-Muurolene	1.00 ± 0.01	1.00 ± 0.01
*δ*-Cadinene	1.00 ± 0.01	
Caryophyllene	1.00 ± 0.01	
*α*-Caryophyllene	0.98 ± 0.01	
***Oxides***		
Caryophyllene epoxide	1.53 ± 0.02	1.53 ± 0.02
***Aromatic hydrocarbons***		
Naphthalene, 1,2-dihydro-1,1,6-trimethyl-	1.00 ± 0.01	1.00 ± 0.01

### Antioxidant activity

Antioxidant activities of EOs were evaluated by three tests (DPPH radical scavenging test, *β*-carotene bleaching (BCB) test, and thiobarbituric acid reactive species (TBARS) assay), and three tests referred to our previous experimental process [23].

### Statistical analysis

All experiments were performed in five replicates, and the data were expressed as mean values ± standard deviation. The analysis of variance (ANOVA) was used in the statistical analysis of the data and was computed in SPSS Statistics 18.0 (SPSS Inc., Shanghai, China) software, and a probability value of *P* < 0.05 was considered to represent a statistically significant difference among mean values. Principal component analysis was calculated in Origin Pro 2016 (OriginLab Corporation, USA) software.

## Results and discussion

### Hydrodistillation

In the general hydrodistillation process, EO should float on the surface of aqueous phase and be collected in Clevenger apparatus (Fig 1A) [19]. Unfortunately, EO was not collected on the surface of aqueous phase, while a white volatile matter (WV) and an aqueous phase (AP) were observed in the condenser and collecting tube. In the previous study, there was a large number of high purity *l*-borneol on the inner wall of the condenser in hydrodistillation [22]. As seen from Table 2, the mean yields of WV and the extract of aqueous phase (EAP) were 0.58% and 0.80% (w/w), respectively. The sum of two yields was 1.38% (w/w) and was less than the yield of total volatiles (TV, 2.42%, w/w). Thus, the volatiles of Luodian *B*. *balsamifera* leaves did not entirely exist in the collecting tube of Clevenger apparatus.

**Table 2:** Yields of *l*-borneol and xanthoxylin in the different volatiles from the leaves of Luodian *Blumea balsamifera*.

Extracts	Volatiles^a^ (g)	Yield^b^ (%)	Concentration^c^ (mg/mL)	*l*-Borneol			Xanthoxylin		
				Content^d^ (%)	Weight^e^ (g)	Yield^f^ (%)	Content (%)	Weight (g)	Yield (%)
TV	1.21 ± 0.03	2.42 ± 0.03	-	40.92 ± 0.83	0.50 ± 0.01	0.99 ± 0.02	44.92 ± 0.51	0.54 ± 0.01	1.09 ± 0.02
WV	0.29 ± 0.01	0.58 ± 0.01	-	90.81 ± 2.42	0.26 ± 0.01	0.53 ± 0.02	0.42 ± 0.01	0.001 ± 0.0001	0.002 ± 0.0001
EAP	0.40 ± 0.01	0.80 ± 0.01	80.00 ± 1.90	17.82 ± 0.16	0.07 ± 0.002	0.14 ± 0.002	34.96 ± 0.46	0.14 ± 0.003	0.28 ± 0.01
AF	0.58 ± 0.02	1.16 ± 0.02	-	68.12 ± 1.34	0.40 ± 0.01	0.79 ± 0.02	22.03 ± 0.38	0.13 ± 0.002	0.26 ± 0.01

^a^ Weights of volatiles were extracted from TV, WV, EAP or AF;

^b^ Yields were the weight percentages of volatiles collected from the leaves to the total weight of the leaves;

^c^ Concentration was the extract of aqueous phase to the volume of aqueous phase;

^d^ Content was the weight percentage of *l*-borneol (or xanthoxylin) in TV, WV, EAP or AF;

^e^ Weights of *l*-borneol (or xanthoxylin);

^f^ Yield was the weight percentage of *l*-borneol (or xanthoxylin) to the total weight of the leaves;

TV, total volatiles were extracted for 10 h from the leaves by hydrodistillation and solvent extraction;

WV, white volatiles were on the surface of condenser inner wall in Clevenger apparatus;

EAP, the volatiles were extracted from the aqueous phase in Clevenger apparatus;

AF, “Aifen” was extracted from the first 500 mL distillate.

Qualitative and quantitative analyses of WV and EAP are shown in Table 3, *l*-borneol (content 90.81%) was the main component of WV. It meant that *l*-borneol was mainly present on the surface of the condenser inner wall of the glassware (Clevenger apparatus), and it had little effect on the extraction of EO. Xanthoxylin (content 34.96%) and *l*-borneol (content 17.82%) were the key components of EAP, and xanthoxylin was the important component dispersed in AP during the process of general hydrodistillation. In other words, xanthoxylin could affect the collection of EO. Besides, the contents of camphor, *α*-cubenene, and caryophyllene in WV were more than 1%, and the contents of caryophyllene, caryophyllene epoxide, *γ*-eudesmol were above 5% in EAP. The contents of *l*-borneol and xanthoxylin in TV, WV, and EAP were calculated and compared. The sum of *l*-borneol contents in WV (53.54%) and EAP (14.14%) accounted for 67.68% of total *l*-borneol content in leaves. The content of xanthoxylin was rare in WV, and 25.69% of total xanthoxylin was present in EAP. Other parts of total *l*-borneol and xanthoxylin were still kept in the leaves.

**Table 3:** Compounds of different volatiles and essential oils from the leaves of Luodian *Blumea balsamifera*.

No.	RI^a^	RI^b^	RI^c^	RF^d^	Components^e^	TV (%)	WV (%)	EAP (%)	AF (%)	EO1 (%)	EO2 (%)	EO3 (%)	EO4 (%)	EO5 (%)	EO6 (%)	Identification^f^
1	976	976	1096	1.04	*β*-Pinene	-	0.1 ± 0.01	-	-	-	-	-	-	-	-	MS, RI
2	979	979	1456	1.34	1-Octen-3-ol	-	0.3 ± 0.02	-	0.1 ± 0.01	-	-	-	-	-	-	MS, RI
3	995	991	1394	1.34	3-Octanol	-	0.1 ± 0.01	-	-	-	-	-	-	-	-	MS, RI
4	1100	1096	1549	1.31	Linalool	0.1 ± 0.01	0.4 ± 0.01	0.1 ± 0.01	0.2 ± 0.01	tr	0.05 ± 0.01	0.02 ± 0.01	0.04 ± 0.01	0.1 ± 0.01	tr	MS, RI
5	1103	1101	1607	1.31	Hotrienol	-	0.1 ± 0.01	0.03 ± 0.01	-	0.02 ± 0.01	0.06 ± 0.01	0.03 ± 0.01	0.1 ± 0.01	0.02 ± 0.01	0.03 ± 0.01	MS, RI
6	1145	1146	1536	1.29	Camphor	0.2 ± 0.01	1.6 ± 0.10	0.2 ± 0.01	0.5 ± 0.02	0.02 ± 0.01	tr	tr	0.1 ± 0.01	-	-	MS, RI
7	1170	1167	1702	1.30	***l*-Borneol**	40.9 ± 2.1	90.8 ± 3.1	17.8 ± 0.8	68.1 ± 1.2	1.3 ± 0.03	1.2 ± 0.1	1.8 ± 0.1	1.8 ± 0.1	1.6 ± 0.1	1.5 ± 0.10	MS, RI, COI
8	1201	1188	1739	1.26	*α*-Terpineol	-	-	0.1 ± 0.01	-	tr	0.05 ± 0.01	0.03 ± 0.01	0.03 ± 0.01	0.04 ± 0.01	0.02 ± 0.01	MS, RI
9	1228	1229	1828	1.31	(Z)-Carveol	-	-	0.1 ± 0.01	-	-	-	-	-	-	-	MS, RI
10	1254	1252	1854	1.31	Geraniol	-	-	0.1 ± 0.01	-	-	0.03 ± 0.01	0.02 ± 0.01	0.02 ± 0.01	-	-	MS, RI
11	1294	1290	2198	1.31	Thymol	-	-	0.1 ± 0.01	-	-	-	-	-	-	-	MS, RI
12	1342	1338	1480	1.00	***δ*-Elemene**	-	0.7 ± 0.1	0.1 ± 0.01	0.1 ± 0.01	5.7 ± 0.2	4.0 ± 0.2	4.7 ± 0.2	4.2 ± 0.2	4.6 ± 0.3	6.0 ± 0.2	MS, RI
13	1353	1351	1460	1.00	***α*-Cubenene**	0.3 ± 0.01	1.6 ± 0.4	0.2 ± 0.01	0.2 ± 0.01	12.1 ± 0.2	9.5 ± 0.3	10.6 ± 0.3	9.5 ± 0.3	10.9 ± 0.2	8.6 ± 0.3	MS, RI
14	1357	1360	1729	1.00	Silphiperfol-5,7(14)-diene	-	-	-	-	0.04 ± 0.01	0.02 ± 0.01	0.1 ± 0.01	tr	0.02 ± 0.01	0.03 ± 0.01	MS, RI
15	1358	1358	2164	1.31	Eugenol	-	-	0.2 ± 0.01	0.04 ± 0.01	-	-	-	-	-	-	MS, RI
16	1382	1383	1577	1.00	Modheph-2-ene	-	-	-	-	0.2 ± 0.01	-	0.2 ± 0.01	0.2 ± 0.01	0.1 ± 0.01	0.1 ± 0.01	MS, RI
17	1403	1407	1587	1.00	Longifolene	-	-	-	-	0.1 ± 0.01	0.1 ± 0.01	0.2 ± 0.01	0.1 ± 0.01	0.2 ± 0.01	0.1 ± 0.01	MS, RI
18	1411	1408	1573	1.00	Isocaryophyllene	-	-	-	-	0.1 ± 0.01	0.1 ± 0.01	0.5 ± 0.01	0.1 ± 0.01	0.3 ± 0.01	0.2 ± 0.01	MS, RI
19	1412	1409	1534	1.00	*α*-Gurjunene	-	0.1 ± 0.01	0.1 ± 0.01	-	0.6 ± 0.02	0.5 ± 0.02	0.5 ± 0.02	0.5 ± 0.02	0.3 ± 0.01	0.3 ± 0.01	MS, RI
20	1430	1419	1616	1.00	**Caryophyllene**	1.9 ± 0.2	2.9 ± 0.2	6.6 ± 0.2	2.0 ± 0.1	27.1 ± 1.1	37.0 ± 1.8	34.9 ± 1.3	36.4 ± 1.4	27.0 ± 1.2	22.9 ± 1.2	MS, RI
21	1454	1455	1835	1.30	Geranyl acetone	-	-	0.03 ± 0.01	-	0.2 ± 0.01	-	0.02 ± 0.01	tr	0.1 ± 0.01	0.1 ± 0.01	MS, RI
22	1460	1460	1647	0.98	Alloaromadendrene	0.2 ± 0.01	0.1 ± 0.01	1.2 ± 0.03	0.2 ± 0.01	-	-	-	-	-	-	MS, RI
23	1466	1462	1650	1.00	**dehydro-Aromadendrane**	0.2 ± 0.01	0.2 ± 0.01	1.2 ± 0.04	0.2 ± 0.01	4.8 ± 0.1	2.5 ± 0.1	2.6 ± 0.2	2.9 ± 0.2	3.3 ± 0.2	3.6 ± 0.1	MS, RI
24	1481	1477	1681	1.00	*γ*-Muurolene	-	-	-	-	0.1 ± 0.01	-	-	-	-	-	MS, RI
25	1489	1490	1702	1.00	*β*-Selinene	0.1 ± 0.01	0.04 ± 0.01	0.7 ± 0.02	0.1 ± 0.01	0.4 ± 0.01	0.7 ± 0.02	0.9 ± 0.1	1.0 ± 0.02	0.3 ± 0.01	0.4 ± 0.01	MS, RI
26	1493	1498	1767	1.00	*α*-Selinene	-	-	-	-	0.1 ± 0.01	0.1 ± 0.01	0.1 ± 0.01	0.1 ± 0.01	0.1 ± 0.01	0.1 ± 0.01	MS, RI
27	1506	1500	1725	1.00	*α*-Muurolene	-	-	0.2 ± 0.01	-	0.5 ± 0.02	0.3 ± 0.01	0.3 ± 0.01	0.3 ± 0.01	0.4 ± 0.01	0.3 ± 0.01	MS, RI
28	1517	1513	1767	1.00	*γ*-Cadinene	-	-	0.2 ± 0.01	-	0.6 ± 0.02	0.3 ± 0.01	0.3 ± 0.01	0.3 ± 0.01	0.2 ± 0.01	0.3 ± 0.01	MS, RI
29	1519	1523	1757	1.00	*δ*-Cadinene	0.1 ± 0.01	-	0.9 ± 0.01	0.03 ± 0.01	0.7 ± 0.02	0.6 ± 0.01	0.6 ± 0.01	0.6 ± 0.01	0.6 ± 0.01	0.6 ± 0.01	MS, RI
30	1549	1546	1916	1.00	*α*-Calacorene	-	-	-	-	0.1 ± 0.01	0.04 ± 0.01	0.03 ± 0.01	0.04 ± 0.01	0.03 ± 0.01	0.1 ± 0.01	MS, RI
31	1558	1559	2083	1.31	(E)-Dauca-4(11),7-diene	0.1 ± 0.01	-	0.5 ± 0.01	0.1 ± 0.01	0.3 ± 0.01	0.2 ± 0.01	0.3 ± 0.01	0.3 ± 0.01	0.2 ± 0.01	0.2 ± 0.01	MS, RI
32	1569	1563	2006	1.34	Nerolidol	0.1 ± 0.01	-	0.8 ± 0.01	0.1 ± 0.01	0.3 ± 0.01	0.2 ± 0.01	0.2 ± 0.01	0.3 ± 0.01	0.3 ± 0.01	0.2 ± 0.01	MS, RI
33	1572	1572	2028	1.34	Caryolan-8-ol	0.1 ± 0.01	-	0.6 ± 0.01	0.1 ± 0.01	0.5 ± 0.02	0.3 ± 0.01	0.3 ± 0.01	0.3 ± 0.01	0.3 ± 0.01	0.4 ± 0.01	MS, RI
34	1587	1583	1990	1.53	**Caryophyllene epoxide**	1.5 ± 0.01	-	7.7 ± 0.3	1.9 ± 0.1	6.9 ± 0.3	4.2 ± 0.3	3.6 ± 0.2	4.0 ± 0.2	4.7 ± 0.2	5.4 ± 0.2	MS, RI
35	1593	1600	2103	1.31	Guaiol	0.3 ± 0.01	-	2.3 ± 0.1	0.5 ± 0.02	0.8 ± 0.02	0.9 ± 0.02	0.7 ± 0.03	0.9 ± 0.1	1.1 ± 0.02	1.1 ± 0.03	MS, RI
36	1595	1593	2112	1.31	Viridiflorol	0.1 ± 0.01	-	0.8 ± 0.02	0.2 ± 0.01	0.9 ± 0.02	0.2 ± 0.01	0.3 ± 0.01	0.3 ± 0.01	0.5 ± 0.01	0.4 ± 0.01	MS, RI
37	1602	1600	2144	1.31	Rosifoliol	0.1 ± 0.01	-	1.4 ± 0.01	0.3 ± 0.01	0.9 ± 0.03	0.5 ± 0.01	0.5 ± 0.01	0.5 ± 0.02	0.8 ± 0.02	0.8 ± 0.02	MS, RI
38	1622	1619	2119	1.31	**10-*epi*-*γ*-Eudesmol**	0.04 ± 0.01	-	2.5 ± 0.1	0.5 ± 0.02	0.9 ± 0.02	1.2 ± 0.03	0.9 ± 0.1	1.4 ± 0.1	1.3 ± 0.1	1.2 ± 0.1	MS, RI
39	1640	1637	2188	1.30	***γ*-Eudesmol**	1.7 ± 0.2	-	9.4 ± 0.2	0.4 ± 0.01	5.4 ± 0.2	10.7 ± 0.2	8.5 ± 0.2	9.7 ± 0.4	9.4 ± 0.3	15.1 ± 0.9	MS, RI
40	1649	1650	2201	1.30	**Xanthoxylin**	44.9 ± 1.7	0.4 ± 0.02	35.0 ± 1.4	22.0 ± 0.8	11.3 ± 0.3	12.5 ± 0.6	13.6 ± 0.6	13.7 ± 0.9	15.5 ± 0.9	12.0 ± 0.8	MS, RI, COI
41	1655	1650	2220	1.30	***β*-Eudesmol**	0.5 ± 0.02	-	2.4 ± 0.1	0.3 ± 0.01	-	3.0 ± 0.1	3.0 ± 0.1	3.4 ± 0.2	3.7 ± 0.3	4.7 ± 0.2	MS, RI
42	1660	1653	2229	1.31	***α*-Eudesmol**	1.4 ± 0.04	-	-	-	10.6 ± 0.5	3.7 ± 0.2	4.8 ± 0.1	3.5 ± 0.1	3.3 ± 0.1	4.5 ± 0.3	MS, RI
43	1699	1670	2271	1.31	Juniper camphor	-	-	0.1 ± 0.01	-	0.09 ± 0.01	0.2 ± 0.01	0.1 ± 0.01	0.2 ± 0.01	0.03 ± 0.01	0.2 ± 0.01	MS, RI
44	1718	1717	2054	1.30	Pentadecanal	-	-	0.1 ± 0.01	-	0.5 ± 0.02	0.3 ± 0.01	0.2 ± 0.01	0.2 ± 0.01	0.3 ± 0.01	0.6 ± 0.02	MS, RI
45	1726	1723	2378	1.31	(2Z,6E)- Farnesol	-	-	-	-	0.1 ± 0.01	0.1 ± 0.01	0.1 ± 0.01	0.1 ± 0.01	tr	-	MS, RI
46	1749	1754	2312	1.31	Zierone	-	-	-	-	0.1 ± 0.01	0.1 ± 0.01	0.1 ± 0.01	0.1 ± 0.01	-	0.1 ± 0.01	MS, RI
47	1770	1770	2706	1.55	Myristic acid	-	-	0.1 ± 0.01	-	0.04 ± 0.01	0.2 ± 0.01	0.2 ± 0.01	0.2 ± 0.01	0.8 ± 0.02	0.8 ± 0.02	MS, RI
48	1870	1875	1943	1.00	1-Nonadecene	-	-	0.1 ± 0.01	-	0.6 ± 0.01	0.1 ± 0.01	0.1 ± 0.01	0.2 ± 0.01	0.2 ± 0.01	0.2 ± 0.01	MS, RI
49	1873	1869	2805	1.55	Pentadecanoic acid	-	-	0.1 ± 0.01	-	0.1 ± 0.01	0.3 ± 0.02	0.3 ± 0.01	0.2 ± 0.01	0.7 ± 0.01	0.6 ± 0.02	MS, RI
50	1921	1913	2363	1.30	(E,E)-5,9- Farnesyl acetone	-	-	-	-	0.2 ± 0.01	0.1 ± 0.01	0.1 ± 0.01	0.1 ± 0.01	0.1 ± 0.01	0.1 ± 0.01	MS, RI
51	1949	1946	2327	1.31	Isophytol	-	-	-	-	0.2 ± 0.01	-	0.02 ± 0.01	-	-	tr	MS, RI
52	1984	1983	2886	1.55	**Hexadecanoic acid**	-	-	0.7 ± 0.01	0.04 ± 0.01	2.0 ± 0.1	2.6 ± 0.1	2.1 ± 0.1	1.6 ± 0.3	5.5 ± 0.2	4.7 ± 0.1	MS, RI
					Total	95.2 ± 4.5	99.4 ± 4.0	94.4 ± 3.7	97.9 ± 2.5	97.5 ± 3.5	98.5 ± 4.3	98.3 ± 4.0	99.1 ± 4.7	98.8 ± 4.4	98.5 ± 4.8	
					Monoterpene hydrocarbons	-	0.1 ± 0.01	-	-	-	-	-	-	-	-	
					Sesquiterpene hydrocarbons	2.8 ± 0.3	5.6 ± 0.7	11.2 ± 0.7	2.7 ± 0.2	53.8 ± 3.6	56.0 ± 2.7	56.5 ± 2.2	56.3 ± 2.2	48.5 ± 2.0	43.8 ± 1.9	
					Aromatic hydrocarbons	-	-	-	-	0.04 ± 0.01	0.02 ± 0.01	0.1 ± 0.01	tr	0.02 ± 0.01	0.03 ± 0.01	
					Aldehydes	-	-	0.1 ± 0.01	-	0.5 ± 0.02	0.3 ± 0.01	0.2 ± 0.01	0.2 ± 0.01	0.4 ± 0.01	0.6 ± 0.02	
					Alcohols	45.8 ± 2.2	91.6 ± 3.2	39.2 ± 1.5	70.9 ± 1.3	22.4 ± 0.9	22.2 ± 0.7	21.5 ± 0.8	22.5 ± 1.1	22.7 ± 1.0	30.2 ± 1.7	
					Ketones	45.1 ± 1.7	2.1 ± 0.1	35.3 ± 1.5	22.5 ± 0.9	11.9 ± 0.4	12.8 ± 0.6	14.0 ± 0.7	14.1 ± 0.9	15.6 ± 0.9	12.4 ± 0.8	
					Acids	-	-	0.8 ± 0.03	0.04 ± 0.01	2.1 ± 0.1	3.1 ± 0.2	2.6 ± 0.2	2.0 ± 0.3	6.9 ± 0.3	6.1 ± 0.2	
					Oxides	1.5 ± 0.01	-	7.7 ± 0.3	1.9 ± 0.1	6.9 ± 0.3	4.2 ± 0.3	3.6 ± 0.2	4.0 ± 0.2	4.7 ± 0.2	5.4 ± 0.2	

^a^ RIs calculated using a homologous series C_8_-C_30_ *n*-alkanes (DB-5 column);

^b^ RIs reported from NIST standard library mass spectral data and Adam’s records [25];

^c^ RIs calculated against *n*-alkanes C_8_–C_30_ (CP-WAX column);

^d^ Response factors;

^e^ Identification performed by means of comparison of RIs and GC-MS spectra (Wiley and NIST databases);

TV, total volatiles were extracted from 5 L distillate from the leaves by hydrodistillation and solvent extraction;

WV, white volatiles were on the surface of condenser inner wall in Clevenger apparatus;

EAP, the volatiles were extracted from the aqueous phase in Clevenger apparatus;

AF, “Aifen” was extracted from the first 500 mL distillate;

EO1-EO6, six essential oils of the leaves harvested from September 2016 to February 2017 (interval of a month), the modified hydrodistillation using Clevenger apparatus;

tr, traces (≤0.01%);

Percentages are given on the apolar column, and values represent the average of three measurements; concentration of a constituent ± SD, n = 3;

^f^ RI, retention indices; MS, by the comparison of MS with those of the computer mass libraries NIST and WILEY; COI, constituent identity confirmed by co-injection of an authentic sample.

For the failure of extracting EO by the general hydrodistillation, we assumed that one or some volatile components had adverse effects on the extraction process of EO. As seen from Table 3, the melting points of several compounds extracted from TV and AP were lower than 80°C, as a result, these compounds in AP (measured temperature: 85°C) were liquid state in the collecting tube, such as caryophyllene, dehydro-aromadendrane, caryophyllene epoxide, guaiol, 10-*epi*-*γ*-eudesmol, *γ*-eudesmol, *β*-eudesmol, *α*-eudesmol, xanthoxylin, etc. A large number of components in volatiles possessed lower density than water and would float on the surface of aqueous phase. However, the density of xanthoxylin (1.172 g/mL) was higher than water, and xanthoxylin would sink to the bottom of the water. Besides, xanthoxylin was the main component of TV (content 44.92%) and EAP. Moreover, the concentrate of xanthoxylin was at a high level (28 mg/mL) in AP in the collecting tube. According to the physicochemical properties of xanthoxylin, we speculated that xanthoxylin dispersed into AP and made the property of AP similar to organic solution. Low-density components would also follow xanthoxylin disperse into AP rather than float on the surface of AP. The total concentration of volatile components in AP was at a high level (80 mg/mL). Thus, the volatile components and water circulated simultaneously in Clevenger apparatus. The coaction of these volatile components made AP play the role of organic solvent to dissolve EO so that EO could not float on the surface of AP to be collected. In a word, the high content of xanthoxylin was the primary cause of the failure to obtain EO of Luodian *B*. *balsamifera* leaves.

### Isolation of essential oil by the modified hydrodistillation

**Fig 2:**
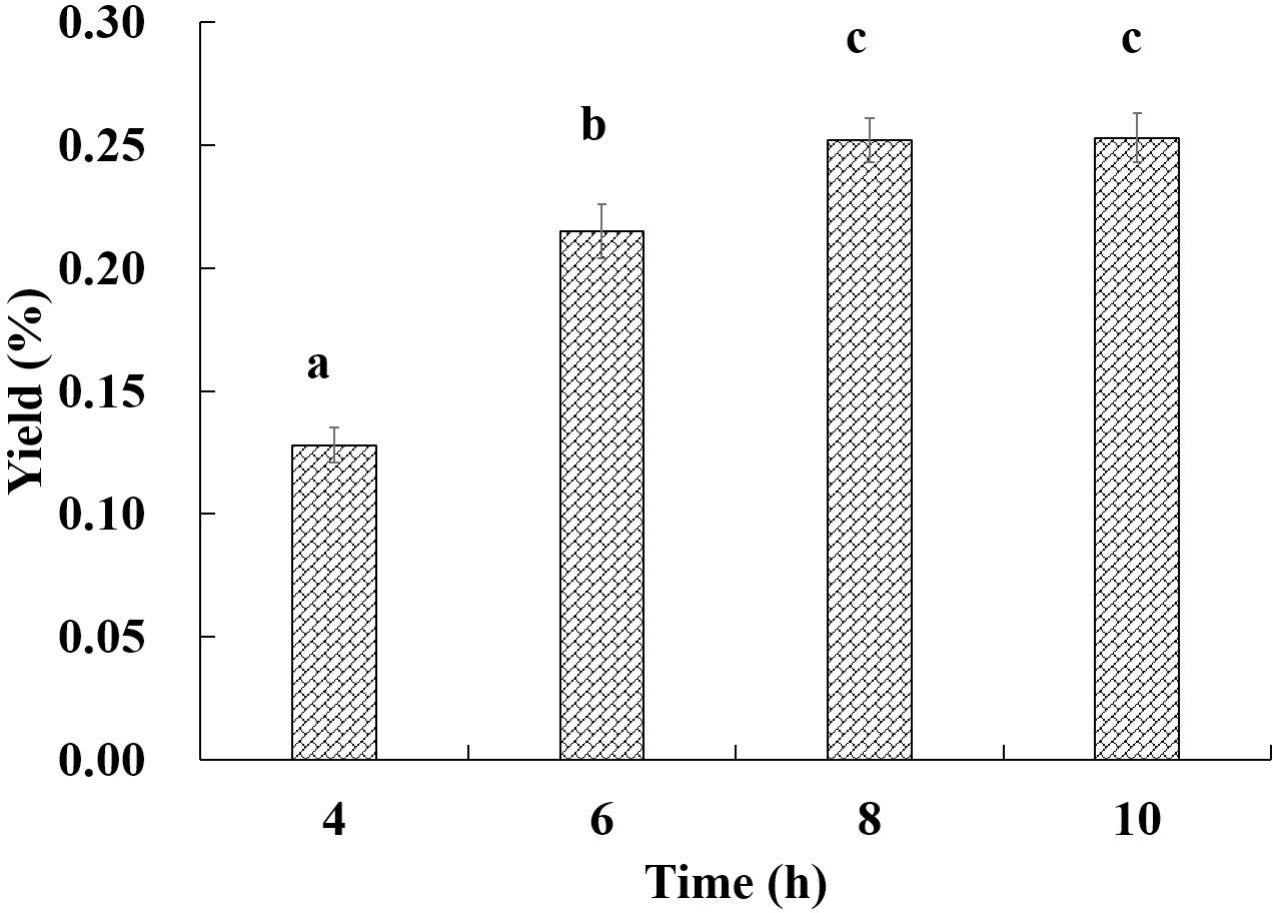
The effect of distillation time on the isolation of essential oil.

**Fig 3:**
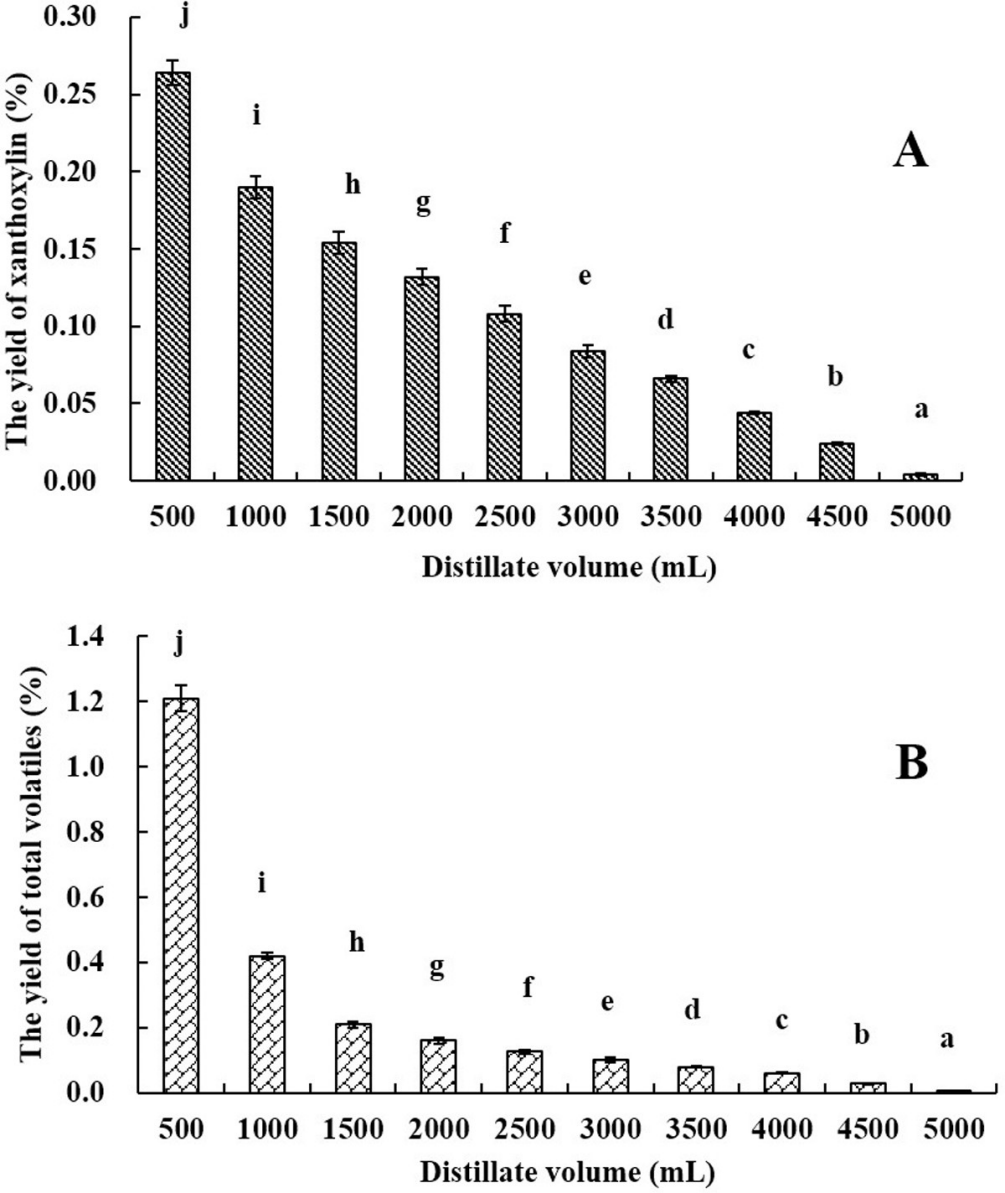
The yields of xanthoxylin (A) and total volatiles (B).

As inferred from the above deduction, if a part of xanthoxylin is removed from the leaves, we designed the modified hydrodistillation procedure to isolate EO of Luodian *B*. *balsamifera* leaves (Fig 1B). Meanwhile, the design also referenced the improved hydrodistillation in the previous study [22,23]. However, the objective of the previous method was the efficient extraction of *l*-borneol and was different from this study. In the beginning, the first 500 mL distillate was distilled to remove partial volatiles (namely “Aifen”) from the leaves, and then the residual leaves were further distilled for collecting EO using Clevenger apparatus. Fortunately, we successfully isolated EO of Luodian *B*. *balsamifera* leaves, and the modified procedure showed that the above deduction was credible. Next, the distillation time was optimized, as seen in Fig 2. The yield of EO did not significantly increase after 8 h. Therefore, eight hours was considered to be the optimal distillation time. As shown in Table 2, the yield of “Aifen” (AF) was 1.16% (w/w), accounted for 47.93% of total volatiles (2.42%, w/w) in leaves (Fig 3B).

It is important to know whether the modified procedure negatively affects the chemical composition of EO or not. Firstly, two key components (*l*-borneol and xanthoxylin) were investigated. *l*-Borneol (content 68.12%) and xanthoxylin (content 22.03%) were two main compositions in AF, and the contents of other volatiles were less (content < 2%). Moreover, *l*-borneol existed in AF was 79.80% of total *l*-borneol in leaves. According to the result of general hydrodistillation, the internal surface of condenser was stained with white volatile matter (WV), and *l*-borneol was the main constituent (content 90.81%) of WV. Because the sublimation property of *l*-borneol, even if *l*-borneol was not distilled and removed in the first 500 mL distillate, *l*-borneol was not in the EO and existed on the surface of condenser inner wall of the glassware.

Besides, the content of xanthoxylin was monitored in the hydrodistillation process. As shown in Fig 3A, the yield of xanthoxylin decreased gradually with the increase of distillation volume, and 23.85% of total xanthoxylin was distilled and removed in the first 500 mL distillate. The above hydrodistillation rule had been confirmed in simultaneous distillation and extraction in the previous study [16]. Therefore, we believed that the removal of partial *l*-borneol and xanthoxylin did not affect the composition of EO. Likewise, the effects of other low content volatile components were little and could be ignored in the collection of EO. Thus, the modified hydrodistillation procedure was feasible to collect EO of Luodian *B*. *balsamifera* leaves.

### Chemical compositions of essential oils

**Fig 4:**
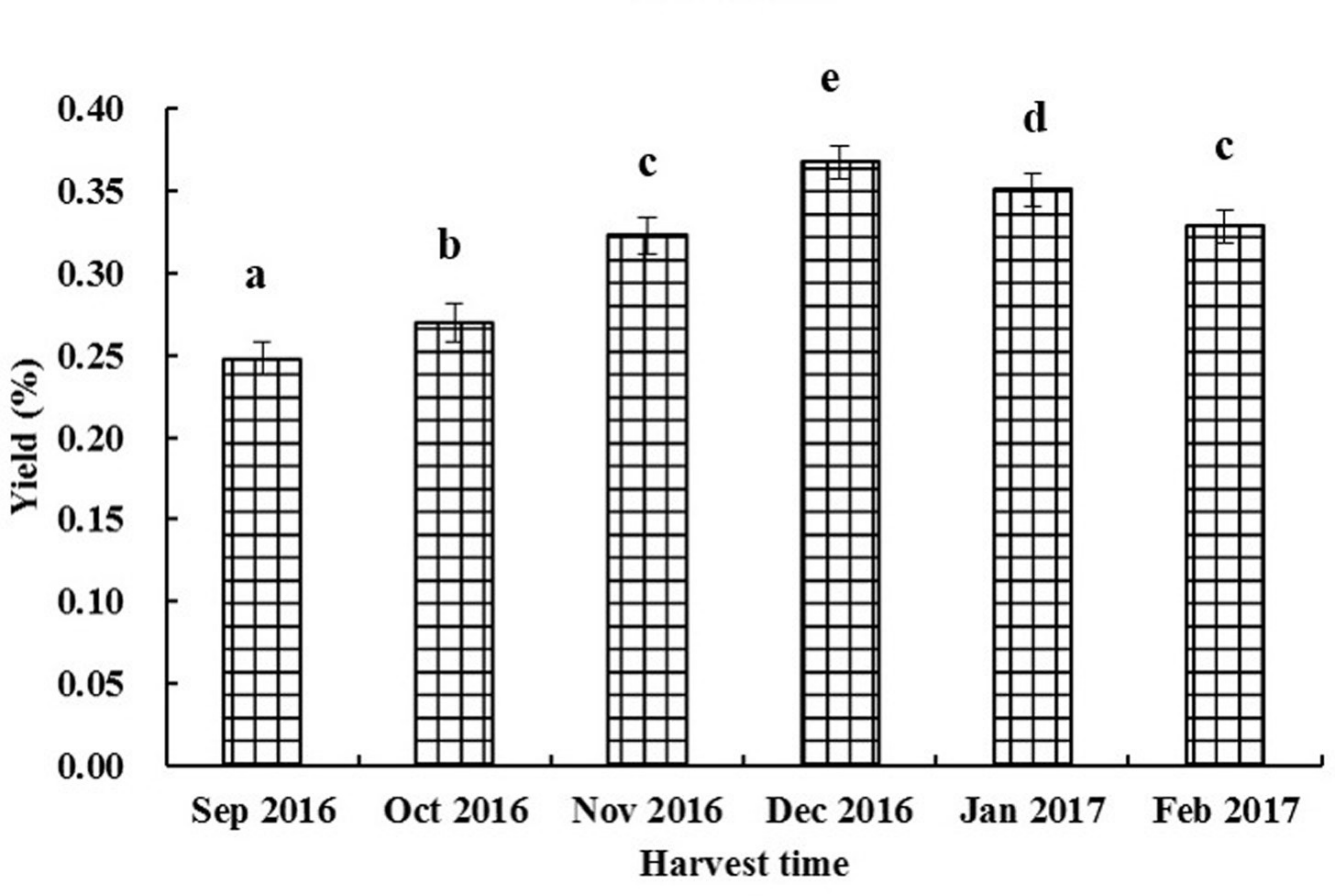
The yields of essential oils of Luodian *B*. *balsamifera* leaves harvested at different times.

Six batches of leaves samples harvested once a month were distilled by the modified hydrodistillation, and all six EOs were collected successfully. The yields of six EOs were 0.25–0.36% (w/w, dry weight) (Fig 4), and the yield increased when the plant grew in the harvest period. The chemical compositions of six EOs (EO1-EO6) are shown in Table 3. Caryophyllene (22.9–36.4%), xanthoxylin (11.3–15.5%), *γ*-eudesmol (5.4–15.1%), and *α*-cubenene (8.6–12.1%) were the main components of six EOs. The other components (>1%) were listed as follow: *l*-borneol, *δ*-elemene, caryophyllene epoxide, 10-*epi*-*γ*-eudesmol, dehydro-Aromadendrane, hexadecanoic acid, *β*-eudesmol, and *α*-eudesmol.

As known from the previous report [15], borneol (57%), caryophyllene (8%), and camphor (5%) are the main constituents in the volatile oil of *B*. *balsamifera* cultivated in Luodian County, China. The main components which exist in the EO of *B*. *balsamifera* grown in Guangxi and Hainan Province, China, Thailand, and Bengal are *l*-borneol (20–42%), caryophyllene (8–10%), camphor (8–20%) [3,4,26]. In previous reports, the contents of xanthoxylin in the EO of *B*. *balsamifera* was 0–4%. Besides, the contents of caryophyllene, *γ*-eudesmol, and α-cubenene are lower. However, in this study, the content of *l*-borneol was very low (only about 1%) in the EO isolated from Luodian *B*. *balsamifera* leaves, and the content of xanthoxylin was 11%.

**Fig 5:**
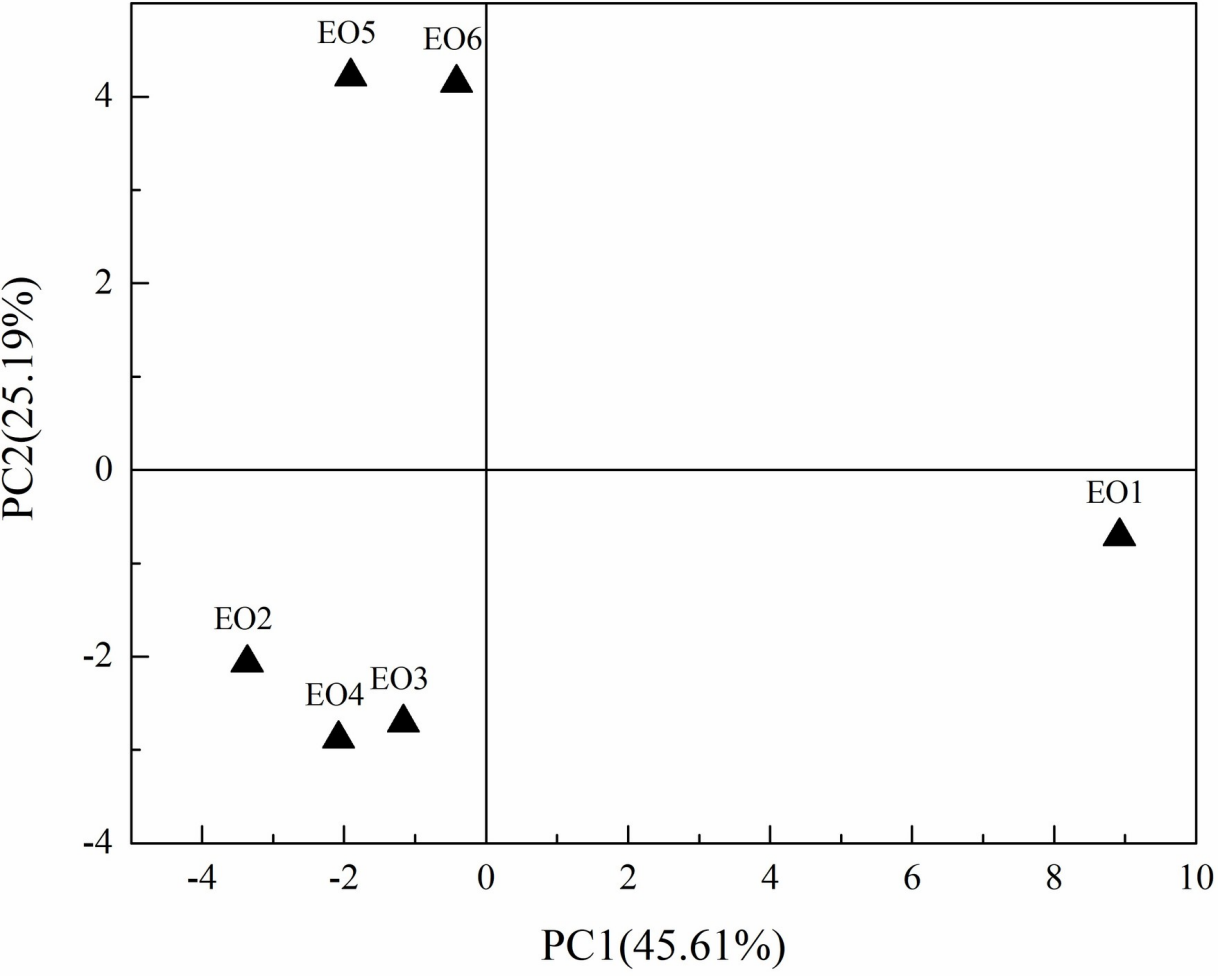
The score-plot of PCA for six EOs of Luodian *B*. *balsamifera*.

To have an overview of all results and to analyse the difference among six EOs harvested at different times. A principal component analysis (PCA) was applied to the data set of six EOs. Fig 5 shows the score-plot for PC1 and PC2 from PCA of different EO samples. PC1 and PC2 explained a great part of the total variance (70.80%). The score-plot was better to understand the relationships between harvest times of the leaves and chemical compounds of EOs. The regions occupied by the six EO samples were distributed in three parts (Fig 5). PCA shows that the chemical components of EO2-EO4 belonged to one category, and were different from that of EO1, EO5, and EO6. It indicated that there were significant differences in chemical components of *B*. *balsamifera* at different growth stages. However, the chemical components of EOs from *B*. *balsamifera* leaves harvested from October to December were comparatively consistent. These results were in agreement with Yuan *et al*., who reported that the component variations of EOs of Hainan *B*. *balsamifera* harvested from October to December (Yuan *et al*., 2016).

### Antioxidant activity

**Fig 6:**
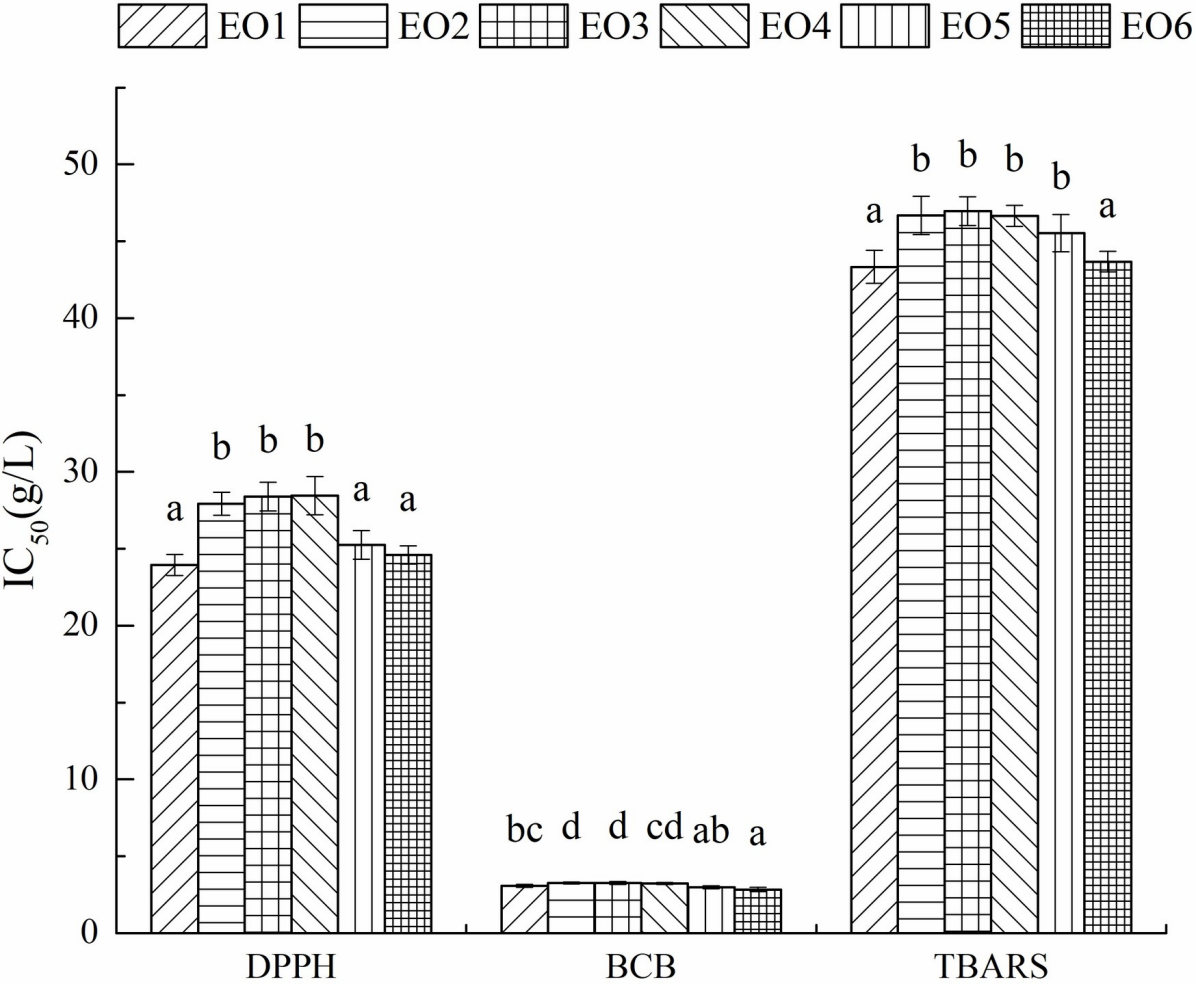
IC_50_ values for antioxidant activities of six EOs.

Antioxidant activities (ACs) of six EOs were evaluated by DPPH radical-scavenging test, *β*-carotene bleaching (BCB) test, and thiobarbituric acid reactive species (TBARS) assay. The results of AC tests are shown in Fig 6. Six EOs exhibited ACs in three tests, which were in agreement with our previous study [23]. Several studies have reported that the ACs of volatile oils and EOs of *B*. *balsamifera* were significantly different in several cultivation areas [4,6,15,17,18,23]. However, to our knowledge, there is no study on the ACs of EOs of Luodian *B*. *balsamifera* in different growth stages. DPPH test, BCB test, and TBRAS test exhibited that the ACs of EO sample No. 2–4 were significantly higher (*P* <0.05). It indicated that the ACs of EOs from the leaves of *B*. *balsamifera* growing from October to December were at a high level. This observation is consistent with previous studies [13]. The joint interpretation of PCA of chemical components and AC tests presented that the AC of EO from *B*. *balsamifera* was highly correlated with the chemical composition of EO. Besides, the yields and the ACs of EOs from *B*. *balsamifera* harvested in November and December were high, and the leaves of *B*. *balsamifera* was suitable for harvesting and extracting EO in these two months.

## Conclusion

The high content of xanthoxylin in the leaves of Luodian *B*. *balsamifera* was considered to be the primary cause of the unsuccessful collection of EO using Clevenger apparatus in the general hydrodistillation. A modified hydrodistillation was designed to isolate EO from Luodian *B*. *balsamifera* leaves. Six EOs of Luodian *B*. *balsamifera* leaves harvested from different growth periods were successfully obtained by the modified hydrodistillation. The EOs contained a considerable amount of caryophyllene and xanthoxylin, which was significantly different from *B*. *balsamifera* cultivated in other regions. The chemical components of EOs from *B*. *balsamifera* leaves harvested from October to December were comparatively consistent, and these EOs had good ACs. Considering the yields and the ACs of EOs, for extracting EO with high quality, Luodian *B*. *balsamifera* leaves should be harvested in November and December. The EO may be used extensively in pharmaceutical, food, and cosmetic industries.

## Supporting information

S1 Data Data set-raw data of tables and figs. (XLSX)
